# Isoform-level gene expression patterns in single-cell RNA-sequencing data

**DOI:** 10.1093/bioinformatics/bty100

**Published:** 2018-02-27

**Authors:** Trung Nghia Vu, Quin F Wills, Krishna R Kalari, Nifang Niu, Liewei Wang, Yudi Pawitan, Mattias Rantalainen

**Affiliations:** 1 Department of Medical Epidemiology and Biostatistics, Karolinska Institutet, Stockholm, Sweden; 2 Novo Nordisk Research Centre Oxford, Oxford, UK; 3 Department of Health Sciences Research; 4 Department of Molecular Pharmacology and Experimental Therapeutics, Mayo Clinic, Rochester, MN, USA

## Abstract

**Motivation:**

RNA sequencing of single cells enables characterization of transcriptional heterogeneity in seemingly homogeneous cell populations. Single-cell sequencing has been applied in a wide range of researches fields. However, few studies have focus on characterization of isoform-level expression patterns at the single-cell level. In this study, we propose and apply a novel method, ISOform-Patterns (ISOP), based on mixture modeling, to characterize the expression patterns of isoform pairs from the same gene in single-cell isoform-level expression data.

**Results:**

We define six principal patterns of isoform expression relationships and describe a method for differential-pattern analysis. We demonstrate ISOP through analysis of single-cell RNA-sequencing data from a breast cancer cell line, with replication in three independent datasets. We assigned the pattern types to each of 16 562 isoform-pairs from 4929 genes. Among those, 26% of the discovered patterns were significant (*P*<0.05), while remaining patterns are possibly effects of transcriptional bursting, drop-out and stochastic biological heterogeneity. Furthermore, 32% of genes discovered through differential-pattern analysis were not detected by differential-expression analysis. Finally, the effects of drop-out events and expression levels of isoforms on ISOP's performances were investigated through simulated datasets. To conclude, ISOP provides a novel approach for characterization of isoform-level preference, commitment and heterogeneity in single-cell RNA-sequencing data.

**Availability and implementation:**

The ISOP method has been implemented as a R package and is available at https://github.com/nghiavtr/ISOP under a GPL-3 license.

**Supplementary information:**

Supplementary data are available at *Bioinformatics* online.

## 1 Introduction

The emergence of single-cell RNA sequencing (scRNAseq) enables characterization of gene expression variability on the single-cell level (Sandberg, 2014; Wang and Navin, 2015). Prior to the advent of scRNAseq, typical gene expression measurements were only possible based on the average expression level over a large number of cells (bulk-cell RNAseq), which effectively excluded the possibility to study gene expression heterogeneity at the single-cell level.

Single-cell sequencing has been applied in a wide range of research areas to date, including studies of circulating tumor cells (Ramsköld *et al.*, 2012; Sandberg, 2014), breast cancer (Aceto *et al.*, 2014), prostate cancer (Cann *et al.*, 2012), transcriptional dynamics (Trapnell *et al.*, 2014), cell cycle (Buettner *et al.*, 2015), tissue heterogeneity (Achim *et al.*, 2015) and cell-to-cell variation in alternative splicing via isoform-level expression analysis (Marinov *et al.*, 2014; Shalek *et al.*, 2013; Velten *et al.*, 2015). Multiple recently published reviews (Hicks and Baslan, 2017; Navin, 2014; Papalexi and Satija, 2017; Rantalainen, 2017; Tang *et al.*, 2011; Wang and Navin, 2015) provide excellent and broad introduction to single-cell sequencing.

Transcriptional isoforms are defined as mRNA molecules of different length and exon composition originating from the same locus, which code for multiple forms of the corresponding protein. Transcriptional isoforms arise as mRNAs are produced from different transcriptional starting sites, terminated at different polyadenylation sites, or as a consequence of alternative splicing (Black, 2003; Matlin *et al.*, 2005). There are numerous studies of alternative splicing in the context of bulk-cell RNAseq, including studies of tissue-level regulation of isoform expression (Wang *et al.*, 2008) and prediction and quantification of alternative isoforms (Richard *et al.*, 2010; Trapnell *et al.*, 2010).

To date, there are relatively few studies published that are focused on characterization of isoform-level expression at the single-cell level. Potentially novel splice junctions were discovered after studying alternative splicing in single cells (Marinov *et al.*, 2014; Tang *et al.*, 2009). Shalek *et al.* (2013) described bimodality in the expression of genes and isoforms in scRNAseq data. The preference of individual cells to express a particular isoform from multiple-isoform genes was also investigated. However, this study was based on a limited dataset with RNAseq data from only 18 cells. In another study (Velten *et al.*, 2015), statistical modeling was applied to characterize 3′ isoform choice variability in single cells via a transcriptome-wide analysis of scRNAseq data from 48 single-cells using BATSeq (Velten *et al.*, 2015), a sequencing methodology with a prominent 3′ end sequencing bias. Welch *et al.* (2016) introduced a statistical model to detect isoform usage that shows significant biological variation through the contrast of variance of isoform ratios to technical noise. Recently, Karlsson and Linnarsson (2017) investigated the diversity of single-cell mRNA in the mouse brain. They discovered an unusual amount of isoform diversity after a conservative definition of isoform was applied.

In this study, we propose a novel method, ISOform-Patterns (ISOP), for analysis and characterization of single-cell isoform-level gene expression data. ISOP enables analysis of single-cell preference, commitment and heterogeneity of isoform level expression. Based on this method, we defined a set of six principal patterns of isoform expression relationships between isoforms from the same gene, including isoform preference, bimodal isoform preference and mutually exclusive expression commitment. We apply ISOP for analysis of scRNAseq data from a breast cancer cell line (MDA-MB-231; *N* = 327 cells), with replication in three independent single-cell datasets, with the aim of systematically characterizing the extent and nature of single-cell isoform-level expression patterns. We then assess to what extent isoform patterns arise randomly due to the distributional properties of single-cell RNA expression, and we also demonstrate how ISOP can be applied for differential isoform pattern (DP) analysis. Finally, we investigate the characteristics of isoform patterns and the performance of the ISOP method in simulated datasets.

## 2 Materials and methods

### 2.1 Datasets

#### 2.1.1 Real datasets

The primary dataset include 384 scRNAseq samples from a triple-negative breast cancer cell line (MDA-MB-231) of which half of the cells were treated with metformin. Specifically, the MDA-MB-231 cells were cultured in ATCC-formulated Leibovitz’s L-15 Medium (Manassas, VA) supplemented with 10% fetal bovine serum (FBS, Atlanta Biologicals, Flowery Branch, GA) and incubated at 37°C without CO2. Cells were plated into six-well plates at a seeding density of 6 × 104 cells/well and were treated with or without 1 mmol/L metformin (Sigma-Aldrich, St. Louis, MO) after 24 h of incubation. Fresh medium and drug were replaced every 24 h. After 5 days of drug treatment, cells were resuspended and single-cells were captured using the Fluidigm C1 system immediately. Two independent cell culture batches were used from which 2 × 96 untreated cells (control) were captured and 2 × 96 treated cells were captured. Furthermore, cells were captured on two different C1 machines in an orthogonal design in relation to the treatment groups. Sequencing libraries were prepared using the standard Fluidigm protocol based on SMARTer chemistry and Illumina Nextera XT. RNA sequencing of 100 bp paired-end reads was carried out on an Illumina HiSeq with 4.9 million reads/cell on average.

The first public dataset consists of 96 cells from HTC116 cell-line extracted from a public dataset (Wu *et al.*, 2014). Single-cells were captured using the Fluidigm C1 system and sequencing libraries for Illumina sequencing were prepared based on SMARTer chemistry and Illumina Nextera XT. The 96 libraries, divided into two pooled samples of 48 libraries were sequenced on two lanes on a Illumina HiSeq, see further details in the original publication (Wu *et al.*, 2014). The second public dataset includes 305 single-cells from a primary human myoblasts (Trapnell *et al.*, 2014) after eliminating samples with debris, without cells and those containing many cells (bulk-cell). Single-cells were captured using the Fluidigm C1 system and sequencing libraries for Illumina sequencing were prepared based on SMARTer chemistry and Illumina Nextera XT. The last public dataset contains 96 single cells from a primary brain tumor of a glioblastoma multiforme patient (patientID SF10282) from a recent study (Müller *et al.*, 2016). Single-cells were captured and libraries prepared on the Fluidigm C1 system, libraries were pooled for 96-plex sequencing, and sequencing was performed on HiSeq 2500 (Illumina). Further details of the sequence preparation and processing are referred to the original publications.

#### 2.1.2 Simulated datasets

To determine the performance of ISOP and to further investigate the characteristics of isoform patterns, we generated simulated single-cell datasets with predefined distributional properties, which were analysed using ISOP. Two simulated datasets were generated: (i) *scSim*, a dataset of isoforms simulated from the whole transcriptome using the *beta*-Poisson model (Vu *et al.*, 2016) and (ii) *ipSim*, a dataset of isoform pairs simulated at different levels of expression and sparsity.

The *scSim* dataset contains data from 200 cells equally divided into two groups: a control group and a treated group. A simulated biological effect was generated as differential expression (DE) between two groups in 1% of the isoforms, all isoforms pairs were otherwise simulated to be expressed independently of each other. In the *ipSim* dataset, we investigate two scenarios of expression relationships (expression type) between pairs of isoforms: non-differential expression and DE, in addition to exploring different expression levels and degrees of sparsity. For convenience, we annotate a particular simulation case by ‘*X*-*Y*’, where *X* and *Y* are the levels of median expression (in log2 scale of read count of cells with non-zero expression) of isoforms a and b, respectively. In particular, the dataset includes seven levels of equivalent expression of two isoforms: 4–4, 5–5, 6–6, 7–7, 8–8, 9–9 and 10–10 and five types of DE between the two isoforms: 7–6 and 7–8 for 2-fold changes, 7–5 and 7–9 for 4-fold changes and 5–10 for the largest fold changes. In each case of X-Y, 11 levels of sparsity of isoforms are taken into account including 5%, 10% to 90% and 95%. Thus, there are 121 simulation parameter settings defined by the combination of expression type and sparsity levels. Data were simulated 100 times under each parameter setting and results were collected for downstream analyses. Further details about the generation of the simulated dataset can be found in the Supplementary Material. We applied the same analyses for analyses of the simulated dataset as in the analyses of the real biological single-cell datasets, including isoform-pattern detection, test for non-random isoform pattern and DP test (only for *scSim*).

### 2.2 Data preprocessing

The Fastq files from the primary dataset (MDA-MB-231) for single-cell RNAseq were processed through MAP-RSeq pipeline (Kalari *et al.*, 2014) to assess the quality of reads, which includes determination of cells with no or few reads, assessment of duplicate reads, inspection of gene-body coverage, estimation of distance between paired-end reads and evaluation of sequencing depth. The Fastq files were mapped to human hg19 UCSC annotation reference using Tophat (Trapnell *et al.*, 2009) and Bowtie (Langmead *et al.*, 2009) to create bam files. In all further analyses, we used the same type of annotation reference downloaded from igenomes http://support.illumina.com/sequencing/sequencing_software/igenome.html. In practice, only annotation information of chromosomes chr1 to chr22, chrX and chrY were used to quantify isoform- and gene-level expression. Cufflinks (Trapnell *et al.*, 2010) version 2.2.1 (quantification-only mode) was applied to estimate the abundances of gene and isoform expression from the bam files. For the public datasets, we also applied Tophat (Trapnell *et al.*, 2009) and Bowtie (Langmead *et al.*, 2009) with the same annotation reference for read alignments, followed by application of Cufflinks (Trapnell *et al.*, 2010) for quantification of isoform level expression values.

The MDA-MB-231 dataset was subsequently preprocessed further by excluding 57 samples corresponding to empty wells (39 samples) and atypical samples (18 outliers), which were identified by principal component analysis. We also used SCell software (Diaz *et al.*, 2016) for double-checking the initial quality control (Supplementary Fig. S1) and achieved 97% concordance in respect to QC ‘passed’ cells. All cells from the remaining 3% contained high coverage (>0.999) estimated by Good-Turing statistics (Supplementary Fig. S1c) and/or high Gini-Simpson index (Supplementary Fig. S1d). In addition, isoforms expressed in less than 1% of samples were excluded from the further analysis. Isoforms were considered as expressed if their read counts were ≥ 3 in a cell. After pre-processing the isoform-level dataset contained 21 728 isoforms, of which 13 863 isoforms from 4929 genes were multiple-isoform genes, and scRNAseq data from 327 single-cells were available for analysis, of which 162, nearly a half of samples, were from the metformin treated group. Gene-level expression data in this dataset comprised 13 073 genes that were also estimated by application of Cufflinks. The HTC116 dataset, the myoblast dataset and the brain dataset consisted of 26 723, 25 623 and 27 124 isoforms and 5874, 5708 and 5949 multiple-isoform genes, respectively.

### 2.3 Patterns of isoform expression

To characterize isoform-level gene-expression patterns in scRNAseq data and to detect potential subpopulations of cells, log expression differences $\Delta_{i,j,a,b}$ [Equation (1)] between pairs of isoforms {a,b} of a single gene (*j*) at cell *i* from a population of *N* cells (i=1..N) were modeled using a Gaussian mixture model approach [Equations (2) and (3)]. Where $y_{i,j,a}$ and $y_{i,j,b}$ represent the log expression of isoforms *a* and *b* in cell *i*, parameter *w_k_* is the mixing weight for component *k* in the model and *K* is the total number of components in the model. In our analyses, *K* was constrained to ≤3. For simplicity, indexes relating to gene (*j*) and cell (*i*) were omitted from Equations (2) and (3)

(1) $$\Delta_{i,j,a,b}=y_{i,j,a}-y_{i,j,b},$$

(2) $$f(\Delta_{a,b})=\sum_{k=1}^{K}w_{k}N(\Delta_{a,b}|\mu_{k},\sigma_{k}),$$

(3) $$\sum_{k=1}^{K}w_{k}=1$$

Model selection to determine the number of components of each mixture model was based on the Akaike information criterion scores with the additional requirement that the smallest weight $\left(w_{k}\right)$ had to be > 0.025, and that the standard deviation of all components was greater than 0.01. Mixture models were fitted using a computationally efficient histogram-based method implemented in the OCplus package (Pawitan *et al.*, 2005). Fitting of the mixture models using the OCplus algorithm reduces data to a histogram defined by equally spaced bins weighted by the number of data points in each bin, here the number of bins was set to the square root of the number of data points (number of cells). Based on the mixture model approach, we define six principal isoform-pair patterns (see Section 3). The method, ISOP, was implemented in the R package ISOP (version 0.99.1 was used in the analyses in this study), available at (https://github.com/nghiavtr/ISOP) under a GPL-3 license.

### 2.4 Test for non-randomness of isoform-pair distributions

To test if an isoform pair distribution was significantly non-random, we employed a permutation-based approach. For an isoform pair {a,b}, we permuted the isoform vectors and calculate $\Delta_{a,b,\text{perm}}$, which is the expression difference between the permuted isoforms *a* and *b* vectors, 10 000 permutations were applied. Next, we estimated the mean, $E(\Delta_{a,b,\text{perm}})$, from the permutations and for each bin. The permutation-based null distribution was derived from the $\chi^{2}$ goodness-of-fit test of *k*th permuted isoform pair $\Delta_{a,b,\text{perm}}(k)$ and $E(\Delta_{a,b,\text{perm}})$. The observed test statistic was derived from the $\chi^{2}$ goodness-of-fit test between $\Delta_{a,b}$ and $E(\Delta_{a,b,\text{perm}})$. Finally, a *P*-value was computed by comparing the observed test statistic with the permutation-based null distribution of the $\chi^{2}$ statistic. To determine significant (non-random) isoform-pairs, *P*-values were adjusted to account for multiple testing using the method described by Benjamini and Hochberg(1995).

### 2.5 DP analysis

We test whether a treatment effect (metformin exposure) was associated with the probability of cells of being clustered into a particular mixture model component in the isoform pattern models, which would suggest a treatment effect on the distribution of isoform pairs. For each mixture model with more than one component, we assigned individual cells to components (cluster labels) based on the estimate of the probability that cell *i* belongs to component *k*. Subsequently, we applied a permutation test to test the association between the cluster labels and the metformin treatment status. In the permutation test, we permuted the metformin treatment factor (10 000 permutations). Next, we established a null-distribution from the χ^2^ statistics of χ^2^ test between the cluster labels and the permuted group labels. Finally, we computed the χ^2^ statistic of χ^2^ test between cluster labels and the true group labels and compared it to the permutation-based null distribution to obtain a permutation-based *P*-value. The *P*-values were adjusted for multiple testing using the method described by Benjamini and Hochberg (1995).

## 3 Results

We developed and applied a method (ISOP) for transcriptome-wide analysis of the co-variability of expression levels in pairs of isoforms (*a* and *b*) from the same gene in scRNAseq data. ISOP utilizes a Gaussian mixture model approach to model isoform expression difference $\left(\Delta_{a,b}\right)$ on a log scale (see Section 2 for details). Based on the estimated mixture model parameters, including the number of components and the location of the components, expression patterns of pairs of isoforms in individual genes can be systematically characterized and described by a small set of principal isoform expression patterns. The isoform expression patterns can be interpreted in terms of single-cell isoform expression preference, commitment and heterogeneity.

### 3.1 Principal patterns

**Fig. 1.**
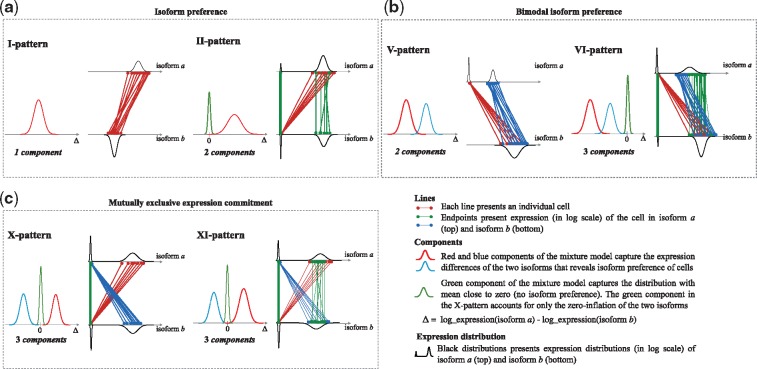
Overview of the six principal isoform expression pattern types. Each panel consists of two plots: a component plot (left) displaying the typical mixture model of $\Delta_{a,b}$ for the pattern, corresponding to isoforms *a* and *b* in the isoform pair, and a pair-line plot (right) of the two isoforms. (**a**) The I-pattern (isoform preference of cells) and its extension, the II-pattern (isoform preference in a subset of cells). (**b**) The V-pattern (bimodal isoform preference of cells) and its extension, the VI-pattern (bimodal isoform preference in a subset of cells). (**c**) The X-pattern (mutually exclusive expression commitment of cells) and its extension, the VI-pattern (mutually exclusive expression commitment in a subset of cells)

Based on ISOP, we define six distinct patterns of isoform expression (Fig. 1) with the following characteristics:

- ***I-pattern***: A single-component model defines this pattern. Thus, there is no cell-to-cell heterogeneity in the isoform pair. However, if the mean of the mixture component is distant from zero, this indicates that one isoform is preferred over the other isoform (preference).
- ***II-pattern***: This pattern is an extension of the I-pattern with an additional mixture component capturing the zero-inflation of cells where isoform expression is not detected, or where isoforms are expressed at close to equal amounts in both isoforms. The II-patterns represents isoform preference in a subset of cells.
- ***V-pattern***: A two-component mixture model defines the V-pattern, in which the means of the two components share the same sign. However, unlike the I-pattern, which has a unimodal distribution in both isoforms, the V-pattern generally has a unimodal expression in one of the isoforms and a bimodal distribution in the other isoform. Thus, the V-pattern is defined by a bimodal isoform preference that indicates cell-to-cell heterogeneity caused by prominent bimodality in one of the two isoforms.
- ***VI-pattern***: This pattern is an extension of the V-pattern with an additional component in the mixture model with its mean close to zero (in analogy to how the II-pattern extends the I-pattern). Thus, the VI-pattern represents the bimodal isoform preference of a subset of cells in population.
- ***X-pattern***: A three-component mixture model defines the X-pattern. Different from the previous pattern types, the X-pattern has two components with the location parameters (mean) of opposite sign and a third component accounting for the zero-inflation. This pattern captures pairs of isoforms with mutually exclusive expression [mutually exclusive isoforms (MXIs)], with similarities to the concept of mutually exclusive exons (MXEs; Wang *et al.*, 2008). The X-pattern represents a mutually exclusive expression commitment that can be interpreted as an indication of commitment of individual cells to express either one of the isoforms, but not both, representing a particular type of inter-cell heterogeneity.
- ***XI-pattern***: This is an extension to the X-pattern where the component located close to zero accounts for both zero-inflation and cells where the two isoforms are expressed at close to equal. Thus, the XI-pattern represents a mutually exclusive expression commitment in a subset of cells in the cell population.

### 3.2 Classification and observed frequencies of isoform patterns

**Fig. 2.**
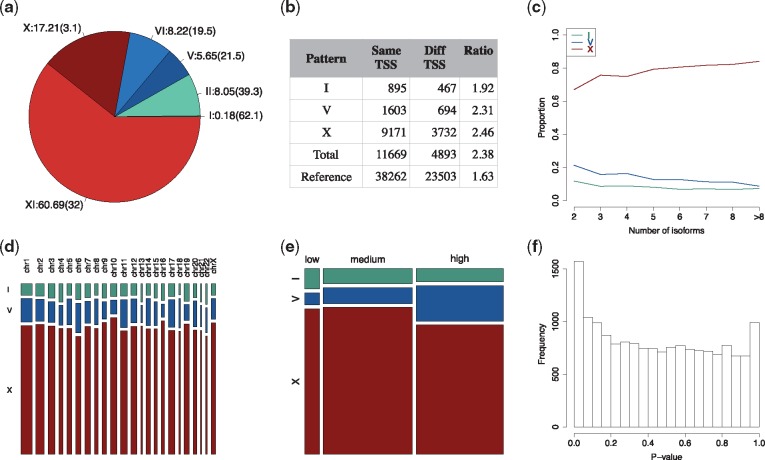
ISOP analysis of the MDA-MB-231 dataset. (**a**) Proportion of isoform patterns and % of isoform patterns within each category that are significant (in parentheses). (**b**) Frequency of patterns with isoforms with the same and different transcription start site (TSS). (**c**) Proportion of patterns as a function of the total number of annotated isoforms in the corresponding gene. (**d**) Proportion of patterns stratified by chromosome. (**e**) Proportion of patterns stratified by gene expression level. (**f**) P-value distribution from the test of association between component label and treatment group

**Fig. 3.**
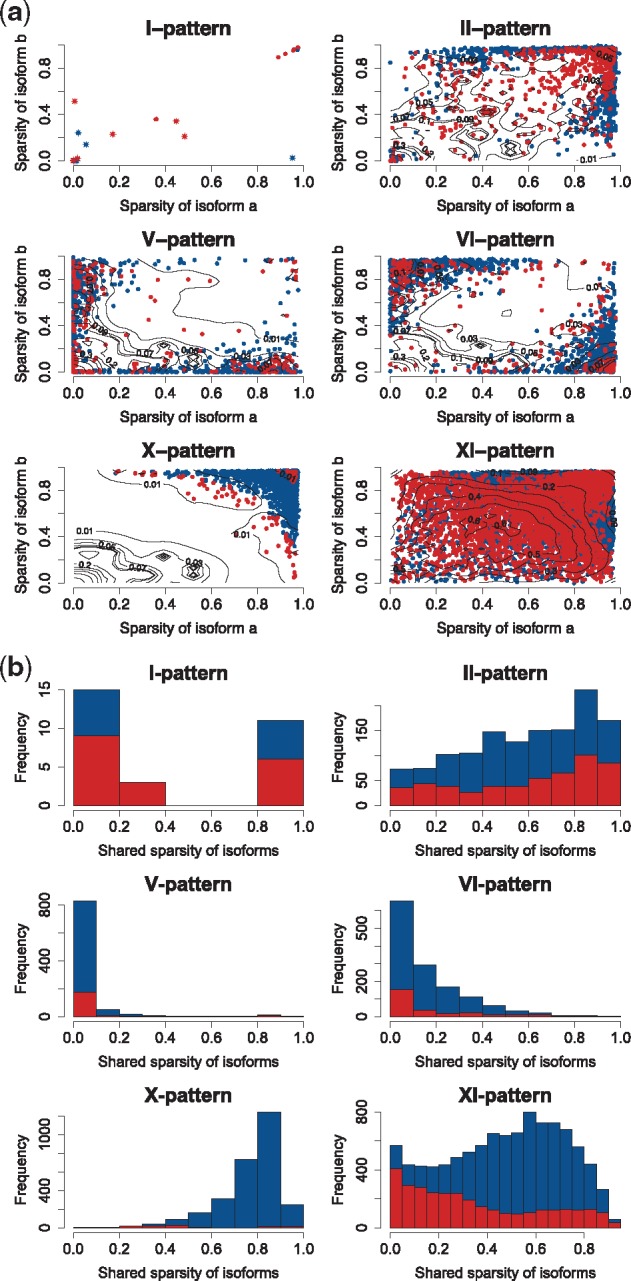
Sparsity of isoform expression in principal isoform expression pattern types. (**a**) Pairwise sparsity of isoform a and b in each individual pair and pattern type. Each point presents a single isoform-pair pattern, while the sparsity of the two isoforms in the pair is indicated on the x- and the y-axes. Blue points and red points represent non-significant and significant isoform-pairs. The star points indicate I-patterns with isoform preference. The contour line presents the two-dimension densities of non-significant isoform pairs using the density smooth function of OCplus package (Ploner *et al.*, 2006). The lower density region indicates the region has a high proportion of significant isoform-pairs. (**b**) Empirical distribution of the shared sparsity of the two isoforms (directly related to the sparsity of $\Delta_{a,b}$) in isoform pairs across pattern types. The red part of the histograms corresponds to the portion of significant (non-random) isoform-pairs

We applied ISOP for analysis of 13 863 isoforms from 4929 multiple-isoform genes in the MDA-MB-231 single-cell dataset. We detected and assigned pattern type to 16 562 isoform-pairs (Fig. 2a). We found that 0.2% of the isoform pairs were classified as I-pattern and 8.1% of the pairs were classified as the related II-pattern. More than a half (55.2%) of the I-patterns were consistent with isoform preference, defined by the absolute mean of the mixture component >0.5 on the log scale, marked by stars in the panel I-pattern of Figure 3a. In contrast, the V-pattern and its extension, the VI-pattern, had proportions in a similar range, 5.7% and 8.2%, respectively (13.9% in total). The X- and XI-patterns were the most common patterns, accounting for 77.9% of the isoform pairs, of which 17.2% were MXIs.

We applied a permutation test (see Section 2) to assess to what extent isoform-pair patterns were significant (non-random, adjusted *P*-value ≤ 0.05). We found 4309 (26.0%) significant isoform patterns in total (Table 1). The I- and II-patterns were the least common patterns (Fig. 2a), while they had the highest proportions of significant isoform-pair patterns, 62.1% and 39.3% for I- and II-patterns, respectively. The X-patterns were the second most common pattern (Fig. 2a), while only 3.1% of these were statistically different from the permutation-based null distribution. A total of 3, 213 (32.0%) of the XI-pattern isoform-pairs were found to be significant, representing the most commonly observed significant isoform pattern.

**Table 1.** Patterns with significant (non-random) isoform-pairs with adjusted *P*-value (Adj.pval) ≤ 0.05 in MDA-MB-231 dataset and the public datasets

Patterns		I	II	V	VI	X	XI	Total
MDA-MB-231	Adj.pval ≤ 0.05	18	524	201	265	88	3213	4309
	Total	29	1333	936	1361	2851	10052	16 562
	Percentage (%)	62.1	39.3	21.5	19.5	3.1	32.0	26.0
HTC116	Adj.pval ≤ 0.05	44	312	110	26	17	704	1213
	Total	1589	7195	2832	1059	2635	8402	23 712
	Percentage (%)	2.8	4.3	3.9	2.5	0.6	8.4	5.1
Myoblast	Adj.pval ≤ 0.05	76	1239	214	253	122	3294	5198
	Total	197	6981	989	1525	4039	9032	22 763
	Percentage (%)	38.6	17.7	21.6	16.6	3.0	36.5	22.8
Brain	Adj.pval ≤ 0.05	33	208	61	6	0	239	547
	Total	1793	7053	3136	328	2958	5707	20 975
	Percentage(%)	1.8	2.9	1.9	1.8	0.0	4.2	2.6

Next, we investigated how expression sparsity might induce isoform patterns. We define sparsity of expression as the proportion of cells where expression levels were below detection limit, so that an isoform with low sparsity has detectable expression levels from most cells. We found that the X-pattern was mainly detected in isoform pairs with high sparsity (Fig. 3a), suggesting that the great majority of X-pattern isoform pairs are likely to arise as a consequence of sparsity, which can be caused by, e.g. transcriptional bursting, biological cell-to-cell heterogeneity or due to the sensitivity limitations of scRNAseq (including transcript drop-out effects). Next, we assessed the distribution of the proportion of cells with zero detected reads in both isoforms in a pair, a quantity directly related to the sparsity of $\Delta_{a,b}$, which is the quantity modeled in the ISOP mixture models (Fig. 3b). It is evident that significant patterns (in red color) often consist of two low sparsity isoforms, with the exception of the I- and II-patterns. Overall, 26.0% of all isoform-pairs were found to be significant (non-random), while a large fraction of non-significant isoform-pairs are likely induced by sparsity in the isoform-level expression data.

To replicate these results, we applied the ISOP method for analysis of three additional public datasets (see Section 2) and found that the same patterns were also discovered in these datasets. There were 23 712, 22 763 and 20 975 isoform pairs in the HTC116, myoblast and brain datasets, respectively. Since these datasets are from diverse tissues, which most likely have substantial differences on the molecular level, the proportions of patterns are rather specific to the dataset. However, we note that the observed isoform patterns have some concordances. For example, the proportions of the different principal patterns in these datasets (Supplementary Fig. S2) were similar to the proportions observed in the MDA-MB-231 dataset, where the X- and the XI-patterns were the most common. The HTC116, myoblasts and brain datasets (Table 1) had 5.1, 22.8 and 2.6% significant patterns, respectively. Similar to the results of the MDA-MB-231 dataset, the non-random isoform-pairs that were annotated as X-patterns in both datasets account for the smallest proportion compared to the other patterns, 0.6% in the HTC116 dataset, 3.0% in the myoblast dataset and 0.0% in the brain dataset.

### 3.3 Association of isoform patterns with genomic features

We then assessed if isoform patterns were associated with different transcription start site (TSS), the number of annotated isoforms of the gene, chromosome location and average expression level. In the following analyses, we merge I, V and X patterns with their corresponding extension patterns because the principal and extended patterns represent the same characteristics of the isoform patterns (isoform preference, bimodal isoform preference or mutually exclusive expression commitment). We observed that the number of isoform pairs originating from the same TSS is more than twice as common (2.38 times, across all patterns) compared with pairs originating from different TSS, compared to a ratio of 1.63 times observed in the reference transcriptome (Fig. 2b). Thus, across all detected patterns, isoform pairs from the same TSS are more frequently observed than those originating from different TSS. The number of isoforms in each gene positively correlates with the proportion of the X-pattern (*r* = 0.92, top line), negatively correlates with the proportion of I-pattern and V-pattern (r=−0.82 and r=−0.94, bottom line and middle line) (Fig. 2c). Hence, genes with many isoforms tend to have a higher proportion X-patterns and lower proportion I- and V-pattern. There was no association between pattern type and the chromosome on which the gene is located (Fig. 2d). Next, we investigated to what degree the different isoform patterns were associated with gene-level expression. It is worth noting that the average gene expression has a strong correlation with the variance of gene expression (Supplementary Fig. S3), with higher average expression levels generally has a lower variance. Thus, the average gene expression also reflects the information of the variance of gene expression. We grouped genes into three groups: low, medium and high expression defined by quartiles of average gene expression (<1st quartile, >1th quartile and <3rd quartile, >3rd quartile). Most isoform pairs belonged to the high expression group (45.6%) or the medium expression group (46.8%); while only a small proportion (7.6%) of these are from low expression group (Fig. 2e).

To investigate the relationship between gene features, including TSS, number of annotated isoforms of gene, mean of gene expression and gene length with isoform patterns, we performed multivariate analysis. Particularly, we applied logistic regression to observe the association of X-pattern versus I-pattern (and V-patterns) in the relationship with these gene features. Under the null hypothesis of no association between the gene characteristics and the pattern type, what we observe is a realization of random permutation of the pattern type. This means that we can compare the observed associations—as estimated by the logistic regression—against the reference distribution generated by permuting the pattern type a large number of times. For predictor *i*, the *P*-value for the predictor is computed as *P*-value = number of abs(coefficient of predictor *i* in permuted data) ≥ abs(observed coefficient of predictor *i*) divided by the number of permutations *K*. In practice, we use *K* = 10 000. Note that the validity of the permutation test depends only on the exchangeability of pattern type under the null hypothesis of no association, so we do not assume independence of the isoform pairs. The results (Supplementary Table S1) show that all these gene characteristics have significant associations with pattern types, except TSS, which had no association (*P*-value = 0.181) in X-pattern versus V-pattern.

Finally, we analysed the effects of principal isoforms (Rodriguez *et al.*, 2015), which encodes for the main protein isoforms detected in proteomics experiments on isoform patterns. The information of principal isoforms available from the APPRIS database (Rodriguez *et al.*, 2015) was downloaded and utilized in this study. We found that most of the isoform pairs with a V patterns (59%) included a principal isoform (Supplementary Table S2). This proportion is significantly smaller compared with the I patterns (36%) and X patterns (33%). Thus, the non-principal isoforms tend to have mutually exclusive expression commitment (X patterns).

### 3.4 Isoform patterns provide a novel way to assess biological effects in scRNAseq data

Next, we tested for associations between a treatment (metformin exposure) and proportion of cells in each mixture model component (see Section 2 for details). An association between a treatment effect and the proportion of the cells in each mixture component would indicate an effect of the treatment on the isoform-level expression pattern. Figure 2f displays the distribution of empirical *P*-values from the association tests, suggesting an enrichment of low *P*-values. A total of 80 isoform pairs were defined as significant (adjusted *P*-value ≤ 0.05) from 54 genes. We define these genes as DP genes to distinguish them from DE genes. Details of DE analysis to discover DE genes are provided in the Supplementary Material. Of these isoform pairs, 19 (23.8%) were found to be significant and non-random (adjusted *P*-value ≤ 0.05) in respect to the distribution of $\Delta_{a,b}$, using the previously described permutation test. However, non-significant results in respect to the distribution of $\Delta_{a,b}$ does not provide evidence that excludes the possibility of true treatment effects on the proportion of treated cells in the mixture components in these patterns, only that the overall distribution of $\Delta_{a,b}$ could have occurred by chance or beem induced by, e.g. sparsity. This observation is confirmed again in the simulated datasets in the next section. A total of 37 (68.5%) of the DP genes had at least one of the two isoforms differently expressed and five of these DP genes were from isoform pairs (9.3%) with two DE isoforms. Furthermore, 17 DP genes (31.5%) did not have either of the two isoforms differentially expressed. The DP unique genes were: NABP1, SMN2, BTN3A3, CLIC1, CEP85L, ERLIN2, CDK1, TMEM136, DAZAP2, PMP22, SPECC1, ACTG1, NFIC, TNPO2, NFATC2, CDC45 and BCAP31. We further investigate the functional interpretation of genes and isoforms from pattern types and DPs using gene set enrichment analysis (GSEA). Here, we used the Reactome database (Croft *et al.*, 2011) to discover pathways (gene sets) associated with the DP genes and DE genes separately. The results show that the most significant pathways for both DP and DE genes were related to cell-cycle gene sets such as Mitotic G2-G2/M phases(R) and cell cycle checkpoints(R). These findings are consistent with other studies, which have reported that metformin regulates the cell-cycle functions via inhibiting cell proliferation (Pierotti *et al.*, 2013). It is also marked that CK1 and CDC45 from the 17 DP genes are the cell-cycle genes of the G2/M phases, these genes were not discovered in conventional DE analysis. Thus, the 17 DP genes consolidate the discovered pathways from the DE genes. GSEA analysis was subsequently applied to the sub-groups of DP genes in each pattern type. Since the majority of the DP genes had an X-pattern, the group of X-pattern genes was found to have similar results as in the analysis of all DP genes. Due to small size, the sets of DP genes from the V- and I-patterns do not report significant results. The details of all the resulted gene sets are given in the Supplementary Material.

### 3.5 Isoform pattern analysis in simulated datasets

Next, we investigated the performance of ISOP and the characteristics of isoform patterns through simulated datasets. We first analysed data from the *scSim* dataset with the objective of evaluating to what extent patterns arise randomly, to validate the permutation test and to ascertain if differentially expressed isoforms are captured by the DP test. A total of 38 026 isoform patterns were analysed in the *scSim* dataset. Among those, 853 isoform patterns contained at least one pre-defined DE isoforms from multiple-isoform genes. In total, 264 out of 326 (81%) of DE isoforms were included in the set of isoform patterns. The proportions of the isoform patterns are visualized in Supplementary Figure S2d. Similar to the real datasets, the I- and the XI-patterns were the least common (1.07%) and the most common (42.9%). The proportions of the other patterns vary around the ranges of 10–17%. Supplementary Figure S4 presents the *P*-value distributions from the non-randomness test of real datasets and simulated datasets. As expected under the null (independently expressed isoforms in each isoform pair analysed), the distribution of *P*-values from the simulated dataset is uniformly distributed, indicating that the simulated dataset does not contain an increased presence of non-random patterns, also suggesting that the applied test operates as expected. Comparing to those from real datasets with the significant numbers of non-random patterns (Supplementary Fig. S4), significant isoform patterns in the real datasets indicate (non-random) biological information in the data. Supplementary Figure S5 displays the *P*-value distribution of DP analysis from the simulated dataset. We discovered 256 DP isoform patterns (adjusted *P*-values ≤ 0.05) where 244 (95%) of these contain at least one pre-defined DE isoform. 92 of 326 (28%) pre-defined DE isoforms from multiple-isoform genes were included in DP isoform patterns. Thus, DP analysis will capture biological signals (in this simulation, DE isoforms) with a high specificity in the simulated dataset under the expected false discovery rate. However, other types of biological signals can be capture through DP analysis as well, which is also revealed in the analysis of the real datasets. It is worth noting that the adjusted *P*-values of the non-randomness tests of all the DP isoform patterns are high (from 0.76 to 1.00), indicating none of them passes the non-randomness test.

In the second simulation study, we evaluated the effect of expression and sparsity (zero inflation) on detection of isoform patterns and to what extent the permutation-based test perform as expected under the null. For each expression type of two isoforms in the *ipSim* dataset, we collected the frequencies of the six patterns detected in each case from 100 repetitions to observe the distributions of patterns vs sparsity of isoforms (see the contour maps in Supplementary Figs. S6–S17). Similar to Figure 3, the *x-* and *y*-axes of each contour map indicate the level of sparsity of two isoforms. The heat colours range from red to white expressing the levels of frequency from low (0) to high (100). The results reveal that the I-pattern is rarely emerging in the simulated dataset (only found in case 5–5). The X-pattern consistently locates in the top-right of the figure, and when the sparsity of the two isoforms are higher than 0.50. In general, if two isoforms have similar expression levels, the regions of the patterns slightly change by the increase/decrease of the expression levels (Supplementary Figs. S6–S12). However, the regions of the patterns are significantly distorted if the difference between the expression levels of two isoforms is increased (Supplementary Figs. S13–S17). Thus, these investigations show that even under the null assumption of no biological effects, patterns also arise as a function of stochastic expression patterns and especially the degree of sparsity of the two isoforms. However, the uniform distribution of the *P*-values from non-randomness tests shows that we do not detect significant patterns at a higher rate than expected under the null assumption (Supplementary Fig. S18).

## 4 Discussion and conclusion

A unique property of single-cell transcriptomic profiling is the ability to characterize cell-to-cell heterogeneity in cell populations. Our objective was to investigate cellular heterogeneity in isoform-level gene expression based on scRNAseq profiling. We proposed a novel method, ISOP, using a mixture model to model and categorize isoform pairs into principal isoform expression patterns.

We described six principal patterns of isoform expression, which can be interpreted in terms of isoform preference, bimodal isoform preference and mutually exclusive isoform expression commitment. Each pattern type represents a specific expression relationship between a pair of isoforms from the same gene. The I-pattern characterizes isoform preference in the cell population of one isoform over the other isoform. The V-pattern expresses a bimodal isoform preference indicating cell-to-cell heterogeneity associate with one level of expression of one isoform and two levels of expression of the other isoform in the cell population. The X-pattern describes a mutually exclusive expression commitment pattern of the cells to express either one of the isoforms, but not both. The II-, VI- and XI-patterns are extensions of I-, V- and X-patterns, respectively, where a subset of cells display the pattern. The type of isoform preference of cells reported in previous studies (Shalek *et al.*, 2013; Velten *et al.*, 2015) can be accounted for by the I-pattern, V-pattern or their respective extensions. Isoform commitment, as defined by mutually exclusive isoform expression (the X-pattern and XI-pattern) was the most common patterns observed, assigned to 77.9% of the isoform pairs. We showed that a large proportion (26.0%) of isoform pair patterns were found to be statistically significant (non-random), while remaining patterns (74.0%) might have been stochastically generated, mainly as a function of the sparsity (zero inflation) or the degree of bimodality in the isoform expression distribution. Such sparsity can arise due to transcriptional bursting, biological heterogeneity or transcript drop-out effects or other technical limitations inherit to scRNAseq. The isoform patterns were also not found to be associated with the underlying relative similarities of individual cells, or that the patterns are broadly coherent across cells (see details in section 3 of the Supplementary Document).

We also outlined how the ISOP method can be applied to test for biological effects related to the principal isoform expression patterns, which was represented by a small molecule perturbation effect (metformin exposure) in the primary dataset. DP analysis provides a novel approach to detect isoform-related effects that may not have been discovered through conventional DE analysis. We discovered 54 significant DP genes, of which 31.5% were associated with isoforms that were not DE. Thus, significant DP genes constitute novel information that augments traditional DE analyses.

In addition, we investigated the performances of the ISOP method in the analyses of simulated datasets. It was found that DP analysis in the simulated dataset captured the information of pre-define DE isoforms under expected false discovery rate. In addition to the DE effects, DP analysis will also capture other types of biological effect in isoform level single-cell expression data, which will not be captured by conventional DE analyses. Our simulations also demonstrated that isoform patterns can arise as a consequence of stochastic patterns in respect to expression levels and sparsity levels of isoform pairs. However, the simulation studies confirm that random patterns arising under the null (i.e. no biological effect) does not lead to an inflated rejection of the null hypothesis beyond the expected rate. Thus, significant patterns as indicated by the non-randomness tests in the real datasets (Supplementary Fig. S4) are expected to indicate real biological effects.

Our study has some limitations. First, the analysis is focused on the set of annotated isoforms only and we indirectly assume that annotations are correct. Second, the present study did not have External RNA Control Consortium spike-ins that could be used to establish levels of technical noise in the data. Furthermore, many algorithms have been proposed for quantification of isoform level gene expression from RNAseq data (Bray *et al.*, 2016; Patro *et al.*, 2014; Suo *et al.*, 2014;Trapnell *et al.*, 2010) and are all based on slightly different assumptions. In our analyses, we applied the widely used Cufflink software for isoform expression estimation. Isoform-level gene expression quantification is inherently more challenging than gene level quantification, particularly in scRNAseq analysis where there are limited number of RNAseq reads from each cell, and one would expect a degree of variability associated with the quantification algorithm applied. Supplementary Figure S19 shows the distribution of the isoform patterns are largely similar across different methods; Kallisto (Bray *et al.*, 2016) and Sailfish (Patro *et al.*, 2014) had higher concordances with each other than with Cufflinks. Furthermore, we make the assumption that the expression differences between pairs of isoforms on a log scale can be approximated by a Gaussian mixture model. Finally, in this study, we have focused on modeling pairwise isoforms expression patterns, while commitment of cells in sets with more than two isoforms is also interesting and biologically relevant, something that is of interest to explore further in future studies.

In conclusion, ISOP provides a novel approach for characterizing isoform-level expression in single-cell populations. ISOP also introduces a novel approach to discover DP genes associated with biological effects, which is complementary to conventional analysis of DE. Although isoform expression patterns can arise as a function of sparseness in expression patterns, we found that more than a quarter of the patterns in our dataset were found to be non-random, suggesting common occurrence of isoform-level preference, commitment and heterogeneity in single-cell populations.

## Supplementary Material

Supplementary Data

## References

[bty100-B1] Aceto N. et al (2014) Circulating tumor cell clusters are oligoclonal precursors of breast cancer metastasis. Cell, 158, 1110–1122. Times cited: 1. 25171411 10.1016/j.cell.2014.07.013 PMC4149753

[bty100-B2] Achim K. et al (2015) High-throughput spatial mapping of single-cell RNA-seq data to tissue of origin. Nature Biotechnol., 33, 503–509. Times cited: 1. 25867922 10.1038/nbt.3209

[bty100-B3] Benjamini Y., Hochberg Y. (1995) Controlling the false discovery rate: a practical and powerful approach to multiple testing. J. Roy. Stat. Soc. Ser. B (Methodological), 57, 289–300. Times cited: 3.

[bty100-B4] Black D.L. (2003) Mechanisms of alternative pre-messenger RNA splicing. Ann. Rev. Biochem., 72, 291–336. Times cited: 1. 12626338 10.1146/annurev.biochem.72.121801.161720

[bty100-B5] Bray N.L. et al (2016) Near-optimal probabilistic RNA-seq quantification. Nat. Biotechnol., 34, 525–527. 27043002 10.1038/nbt.3519

[bty100-B6] Buettner F. et al (2015) Computational analysis of cell-to-cell heterogeneity in single-cell RNA-sequencing data reveals hidden subpopulations of cells. Nat. Biotechnol., 33,155–160. Times cited: 1. 25599176 10.1038/nbt.3102

[bty100-B7] Cann G.M. et al (2012) mRNA-Seq of single prostate cancer circulating tumor cells reveals recapitulation of gene expression and pathways found in prostate cancer. PLoS ONE, 7, e49144. Times cited: 1. 23145101 10.1371/journal.pone.0049144 PMC3492322

[bty100-B8] Croft D. et al (2011) Reactome: a database of reactions, pathways and biological processes. Nucl. Acids Res., 39, D691–D697. 21067998 10.1093/nar/gkq1018 PMC3013646

[bty100-B9] Diaz A. et al (2016) SCell: integrated analysis of single-cell RNA-seq data. Bioinformatics (Oxford, England), 32, 2219–2220. 10.1093/bioinformatics/btw201 PMC4937196 27153637

[bty100-B10] Hicks J., Baslan T. (2017) Unravelling biology and shifting paradigms in cancer with single-cell sequencing. Nat. Rev. Cancer, 17, 557. 28835719 10.1038/nrc.2017.58

[bty100-B11] Kalari K.R. et al (2014) MAP-RSeq: Mayo analysis pipeline for RNA sequencing. BMC Bioinformatics, 15, 224. Times cited: 1. 24972667 10.1186/1471-2105-15-224 PMC4228501

[bty100-B12] Karlsson K., Linnarsson S. (2017). Single-cell mRNA isoform diversity in the mouse brain. BMC Genomics, 18,126. Times cited: 1. 28158971 10.1186/s12864-017-3528-6 PMC5291953

[bty100-B13] Langmead B. et al (2009) Ultrafast and memory-efficient alignment of short DNA sequences to the human genome. Genome Biol., 10, R25. Times cited: 2. 19261174 10.1186/gb-2009-10-3-r25 PMC2690996

[bty100-B14] Marinov G.K. et al (2014) From single-cell to cell-pool transcriptomes: stochasticity in gene expression and RNA splicing. Genome Res., 24, 496–510. Times cited: 2. 24299736 10.1101/gr.161034.113 PMC3941114

[bty100-B15] Matlin A.J. et al (2005) Understanding alternative splicing: towards a cellular code. Nat. Rev. Mol. Cell Biol., 6, 386–398. Times cited: 1. 15956978 10.1038/nrm1645

[bty100-B16] Müller S. et al (2016) Single-cell sequencing maps gene expression to mutational phylogenies in PDGF- and EGF-driven gliomas. Mol. Syst. Biol., 12, 889. 27888226 10.15252/msb.20166969 PMC5147052

[bty100-B17] Navin N.E. (2014) Cancer genomics: one cell at a time. Genome Biol., 15, 452. Times cited: 1. 25222669 10.1186/s13059-014-0452-9 PMC4281948

[bty100-B18] Papalexi E., Satija R. (2017) Single-cell RNA sequencing to explore immune cell heterogeneity. Nat. Rev. Immunol., 8,35–45. 10.1038/nri.2017.76 28787399

[bty100-B19] Patro R. et al (2014) Sailfish enables alignment-free isoform quantification from RNA-seq reads using lightweight algorithms. Nat. Biotechnol., 32, 462–464. Times cited: 1. 24752080 10.1038/nbt.2862 PMC4077321

[bty100-B20] Pawitan Y. et al (2005) False discovery rate, sensitivity and sample size for microarray studies. Bioinformatics, 21, 3017–3024. Times cited: 1. 15840707 10.1093/bioinformatics/bti448

[bty100-B21] Pierotti M.A. et al (2013) Targeting metabolism for cancer treatment and prevention: metformin, an old drug with multi-faceted effects. Oncogene, 32, 1475–1487. 22665053 10.1038/onc.2012.181

[bty100-B22] Ploner A. et al (2006) Multidimensional local false discovery rate for microarray studies. Bioinformatics, 22, 556–565. 16368770 10.1093/bioinformatics/btk013

[bty100-B23] Ramsköld D. et al (2012) Full-length mRNA-Seq from single-cell levels of RNA and individual circulating tumor cells. Nat. Biotechnol., 30, 777–782. 22820318 10.1038/nbt.2282 PMC3467340

[bty100-B24] Rantalainen M. (2017) Application of single-cell sequencing in human cancer. Brief. Funct. Genomics, https://academic.oup.com/bfg/advance-article/doi/10.1093/bfgp/elx036/4587572. 10.1093/bfgp/elx036 PMC6063300 29106464

[bty100-B25] Richard H. et al (2010) Prediction of alternative isoforms from exon expression levels in RNA-Seq experiments. Nucl. Acids Res., 38, e112. Times cited: 1. 20150413 10.1093/nar/gkq041 PMC2879520

[bty100-B26] Rodriguez J.M. et al (2015) APPRIS WebServer and WebServices. Nucl. Acids Res., 43, W455–W459. 25990727 10.1093/nar/gkv512 PMC4489225

[bty100-B27] Sandberg R. (2014) Entering the era of single-cell transcriptomics in biology and medicine. Nat. Methods, 11, 22–24. Times cited: 2. 24524133 10.1038/nmeth.2764

[bty100-B28] Shalek A.K. et al (2013) Single-cell transcriptomics reveals bimodality in expression and splicing in immune cells. Nature, 498, 236–240. Times cited: 3. 23685454 10.1038/nature12172 PMC3683364

[bty100-B29] Suo C. et al (2014) Joint estimation of isoform expression and isoform-specific read distribution using multisample RNA-Seq data. Bioinformatics (Oxford, England), 30, 506–513. Times cited: 1. 10.1093/bioinformatics/btt704 24307704

[bty100-B30] Tang F. et al (2009) mRNA-Seq whole-transcriptome analysis of a single cell. Nat. Methods, 6, 377–382. Times cited: 1. 19349980 10.1038/nmeth.1315

[bty100-B31] Tang F. et al (2011) Development and applications of single-cell transcriptome analysis. Nat. Methods, 8, S6–S11. Times cited: 1. 10.1038/nmeth.1557 PMC3408593 21451510

[bty100-B32] Trapnell C. et al (2009) TopHat: discovering splice junctions with RNA-Seq. Bioinformatics, 25, 1105–1111. Times cited: 2. 19289445 10.1093/bioinformatics/btp120 PMC2672628

[bty100-B33] Trapnell C. et al (2010) Transcript assembly and quantification by RNA-Seq reveals unannotated transcripts and isoform switching during cell differentiation. Nat. Biotechnol., 28, 511–515. Times cited: 4. 20436464 10.1038/nbt.1621 PMC3146043

[bty100-B34] Trapnell C. et al (2014) The dynamics and regulators of cell fate decisions are revealed by pseudotemporal ordering of single cells. Nat. Biotechnol., 32, 381–386. Times cited: 3. 24658644 10.1038/nbt.2859 PMC4122333

[bty100-B35] Velten L. et al (2015) Single-cell polyadenylation site mapping reveals 3’ isoform choice variability. Mol. Syst. Biol., 11, 812. Times cited: 4. 26040288 10.15252/msb.20156198 PMC4501847

[bty100-B36] Vu T.N. et al (2016) Beta-Poisson model for single-cell RNA-seq data analyses. Bioinformatics, 32,2128–2135. Times cited: 1. 27153638 10.1093/bioinformatics/btw202 PMC13048230

[bty100-B37] Wang E.T. et al (2008) Alternative isoform regulation in human tissue transcriptomes. Nature, 456, 470–476. Times cited: 2. 18978772 10.1038/nature07509 PMC2593745

[bty100-B38] Wang Y., Navin N.E. (2015) Advances and applications of single-cell sequencing technologies. Mol. Cell, 58, 598–609. Times cited: 2. 26000845 10.1016/j.molcel.2015.05.005 PMC4441954

[bty100-B39] Welch J.D. et al (2016) Robust detection of alternative splicing in a population of single cells. Nucl. Acids Res., 44,e73. Times cited: 1. 26740580 10.1093/nar/gkv1525 PMC4856971

[bty100-B40] Wu A.R. et al (2014) Quantitative assessment of single-cell RNA-sequencing methods. Nat. Methods, 11, 41–46. Times cited: 2. 24141493 10.1038/nmeth.2694 PMC4022966

