# Supplementary material for: Isoform-level gene expression patterns in single-cell RNA-sequencing data
Source: Bioinformatics. 2018 Feb 27;34(14):2392–400. doi: 10.1093/bioinformatics/bty100 (PMC6041805; doi:10.1093/bioinformatics/bty100)
Supplement: Supplementary Data [file bty100_supp.zip › bty100-suppl_data/ISOP_supplementary documents_final.docx]

Supplementary documents

Isoform-level gene expression patterns in single-cell RNA-sequencing data

By Trung Nghia Vu, et al

1. **Simulation datasets**

The first simulated single cell dataset (*scSim*) includes two groups control vs treated from beta-Poisson model (Vu *et al.*, 2016), with 100 cells in each group. First, we collect the well fitted four-parameter beta-Poisson model from the HTC116 dataset (Monte-Carlo p-value >= 5%) as the baseline distribution for gene expression. Then for each isoform, the same beta-Poisson model is used to generate the expression across samples in both the control group and the treated group. Finally, we randomly assign 1% (480) isoforms for DE isoforms where 70% of them (326 isoforms) are from multiple-isoform genes. To create the biological effects for the DE isoforms, the parameter λ*­*_1_ of the beta-Poisson model of one group is multiplied by the fold-change (=4.0). We apply the same analyses to the simulated dataset including the isoform-pattern detection, the non-randomness test of isoform pattern and differential pattern

The second simulated dataset (*ipSim*) is investigated in two main different scenarios: variances by expression and variances by sparsity. Seven levels of equivalent expression of isoforms (from low (4-4) to high (10-19) in log2 scale of read counts) are considered. Moreover, five different types of fold-change expression between two isoforms are also investigated: 7-6 and 7-8 for 2 fold-change, 7-5 and 7-9 for 4 fold-change and 5-10 for strong expression different bias. Herein, the first number and the second number of the expression type which are separated by the dash “-“, are the expression (in log2 scale) of the first and the second isoforms in the isoform pair respectively; and the expression of isoform is understood as the median expression of expressed cells of the isoform. In each type of expression, eleven levels of sparsity of isoforms are taken into account: 0.05, 0.1, 0.2… 0.80, 0.90, and 0.95. To simulate the data for each case, we collect all low sparsity isoforms (sparsity level < 0.01) from the simulated dataset (*scSim*) and select two isoforms with the expected median expression. Then, in each isoform, we randomly suppress expression of a number of cells to zero to achieve the expected sparsity level. Finally the pattern detection method is deployed to get the pattern of the isoform pair. We repeat 100 times for each case before extracting results. We collect the numbers of times which six patterns are detected in each case from 100 repetitions to observe the distributions of patterns vs sparsity of isoforms (see the contour maps in Figure S5-S15).

1. **Differential expression analysis**

To identify differentially expressed (DE) isoforms, we applied a linear model as implemented in the limma package version 3.22.1 (Smyth, 2005), accounting for the mean-variance trend in the data, which has also be applied for analysis of RNAseq data (Law *et al.*, 2014). Prior to the differential expression analysis, the count dataset was expressed as log-counts per million (cpm) using functionality in the edgeR package version 3.8.2 (McCarthy *et al.*, 2012). We accounted for culture batch effect and machine effect in the experimental design matrix in the limma analysis of the primary dataset. We applied a permutation test to identify DE isoforms. First, 100 permuted datasets were generated by randomly permuting group labels. Next, the moderated t-statistics (extracted from limma) of isoforms from the actual dataset and the population of the moderated t-statistics of the isoforms from permutated datasets were used to calculate empirical p-values of the isoforms. Finally, the empirical p-values were adjusted by using Benjamini & Hochberg (BH) correction. DE isoforms were defined as isoforms with adjusted p-values less than or equal to 5\%.

1. **Cell clusters from ISOP mixture components**

We did further analysis to investigate the relationship between underlying relative similarities of individual cells with the isoform patterns as follows. First, for each individual isoform pattern, the cells are clustered to the components of its mixture model. Thus, the components of the mixture model represent the groups of cells. Then, the similarity between two individual cells are measured globally by gene expression using Euclidean distance. Next, we evaluate the goodness of the cell clusters of an isoform pattern by the F-test statistic from the ratio between the between- vs within-group variability (of pair-wise Euclidian distances). Finally, the p-values of the F-tests from all isoform patterns are adjusted for multiple-testing using Benjamini & Hochberg method. Based on this analysis, we found that none of the isoform patterns were associated with the global expression patterns (the best adjusted p-values > 0.23). Thus, the isoform patterns were not found to be associated with the underlying relative similarities of individual cells, or that the patterns are broadly coherent across cells.

**TABLES**

Table S1. P-values of multifactorial analysis of isoform patterns vs genomics features using logistics regression. X-pattern is used as the reference in the logistics regression.

| **Patterns** | **I** | **V** |
| --- | --- | --- |
| **TSS** | 1.0e-04 | 1.81e-01 |
| **Number of isoforms** | 1.0e-04 | 1.0e-04 |
| **Mean of gene expression** | 1.0e-04 | 1.0e-04 |
| **Gene length** | 2.0e-04 | 1.0e-04 |

Table S2. Proportions of isoform patterns containing at least one principal isoform.

| **Patterns** | **I** | **V** | **X** | **Total** |
| --- | --- | --- | --- | --- |
| **Principal isoform** | 485 | 1360 | 4264 | 6109 |
| **Total** | 1362 | 2297 | 12903 | 16562 |
| **Percentage** | 35.6% | 59.3% | 33.0% | 36.9% |

Table S3. Distribution of significant isoform patterns from cell-cycle genes across cell-cycle phases and pattern types.

|  | **I** | **V** | **X** | **Proportion (%)** |
| --- | --- | --- | --- | --- |
| **G1/S** | 5 | 1 | 19 | 29.8 |
| **G2** | 0 | 0 | 1 | 1.2 |
| **G2/M** | 9 | 8 | 32 | 58.3 |
| **M/G1** | 1 | 1 | 7 | 10.7 |
| **Proportion (%)** | 17.9 | 11.9 | 70.2 |  |

**FIGURES**


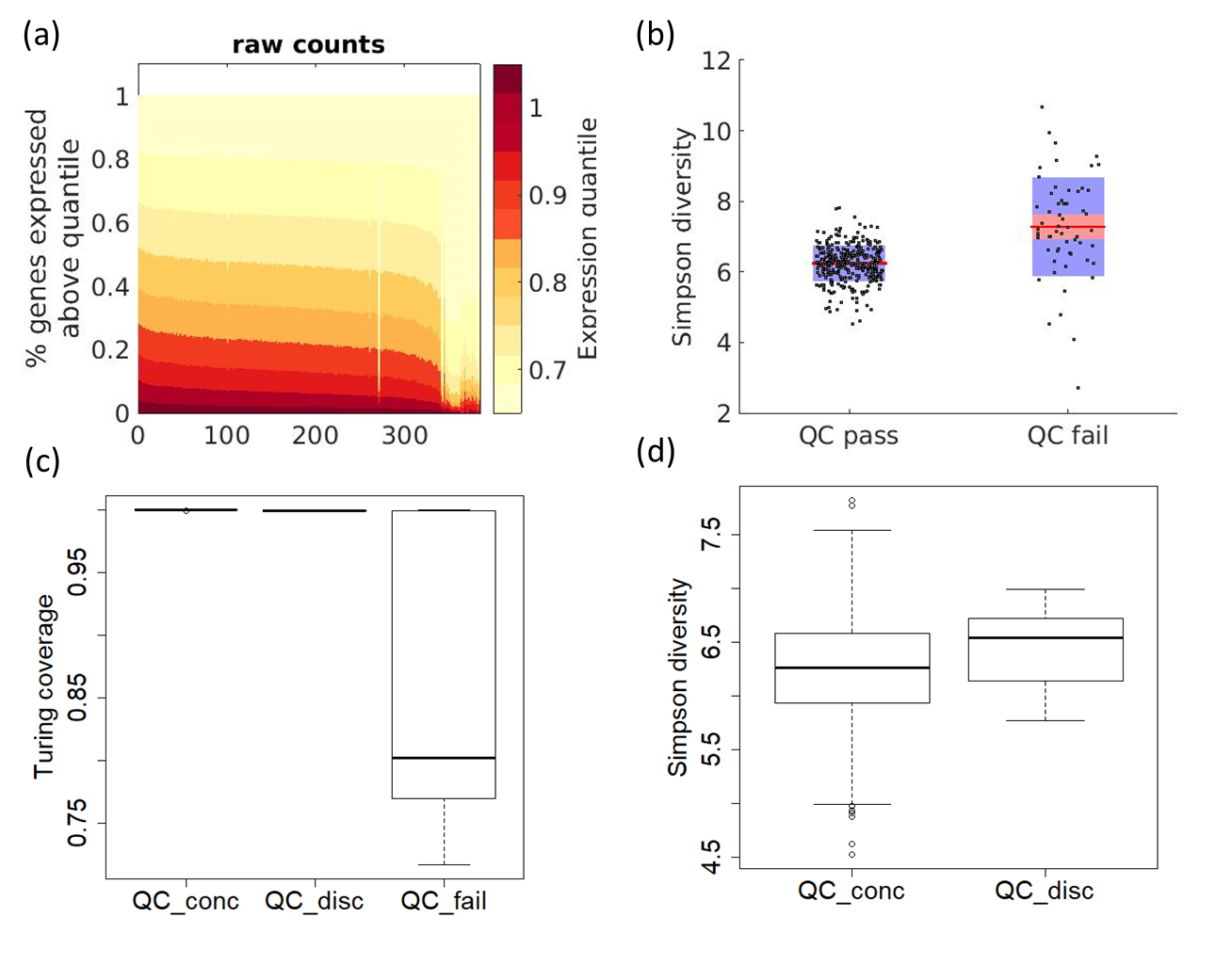


Figure S1. Quality control of the MDA-MB-231 dataset using SCell: a) Percent of genes expressed above a given expression percentile from SCell b) Simpson diversity scores from Scell; c) and d) Turing coverage and Simpson diversity of concordant cells (QC_conc), discordant cells (QC_disc) from SCell quality control.


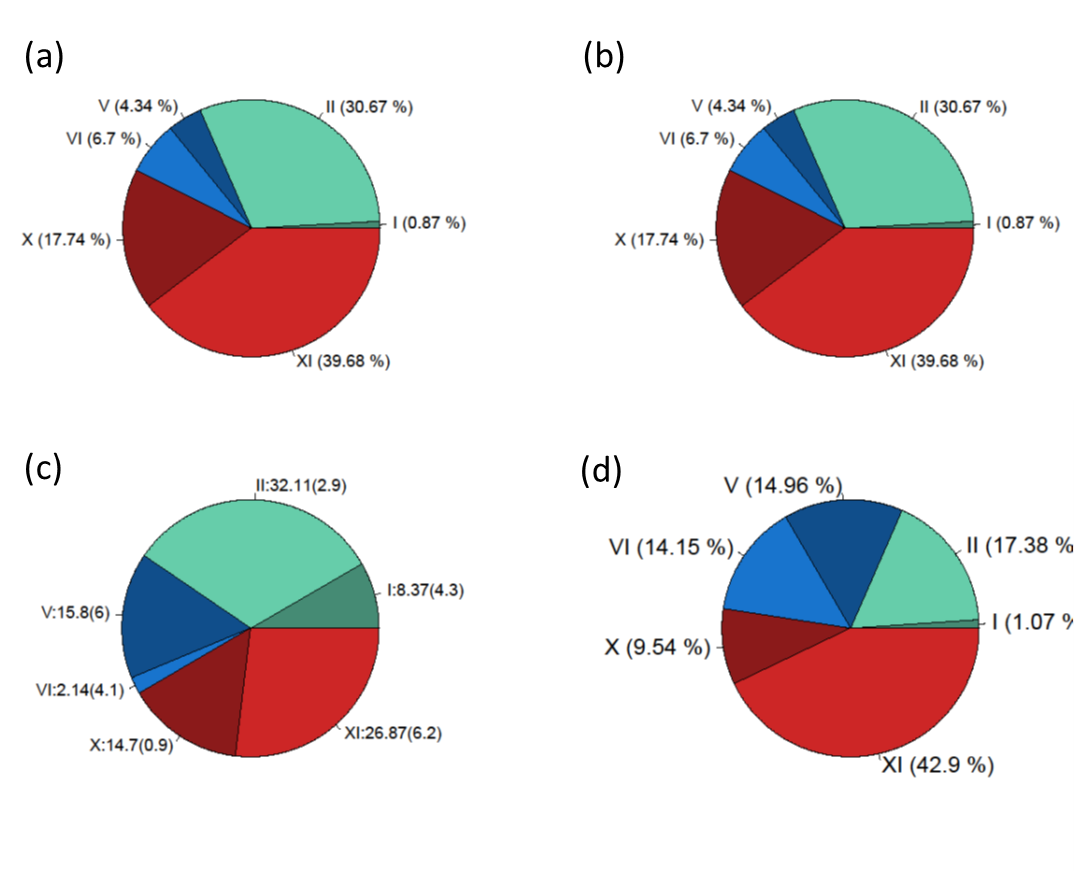


Figure S2: Proportion of patterns detected in replication data sets: (a) HTC116 dataset; (b) myoblast dataset; (c) brain dataset; (d) simulated dataset *scSim.*


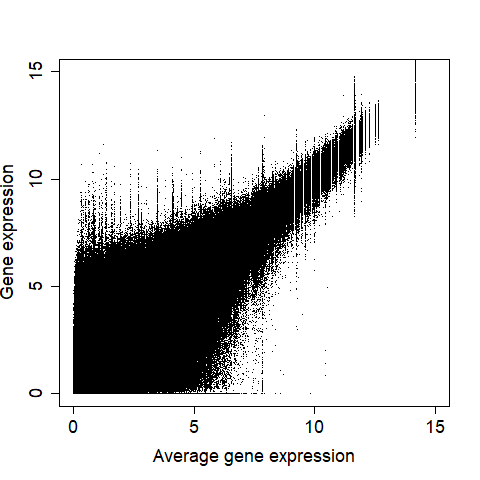


Figure S3: Distribution of gene expression according to the average gene expression from the MDA-MB-231 dataset. The x-axis presents the level of the average gene expression, the y-axis presents the actual expression of the gene. Each dot in the plot present the expression of a gene in an individual cell. The expression is presented in log scale: expression x is transformed to log2(x+1).

MDA-MB-231

HTC116

Myoblast

scSim

Figure S4: P-value distributions of the non-randomness tests from the simulated dataset *scSim* and real datasets

Figure S5: P-value distribution of the differential pattern analysis from the simulated dataset *scSim*.

Figure S6: Distribution of isoform patterns in different sparsity levels of isoforms: **case 4-4** where median expressions of expressed cells of two isoforms are around 4.0 (log2 scale).

Figure S7: Distribution of isoform patterns in different sparsity levels of isoforms: **case 5-5** where median expressions of expressed cells of two isoforms are around 5.0 (log2 scale).

Figure S8: Distribution of isoform patterns in different sparsity levels of isoforms: **case 6-6** where median expressions of expressed cells of two isoforms are around 6.0 (log2 scale).

Figure S9: Distribution of isoform patterns in different sparsity levels of isoforms: **case 7-7** where median expressions of expressed cells of two isoforms are around 7.0 (log2 scale).

Figure S10: Distribution of isoform patterns in different sparsity levels of isoforms: **case 8-8** where median expressions of expressed cells of two isoforms are around 8.0 (log2 scale).

Figure S11: Distribution of isoform patterns in different sparsity levels of isoforms: **case 9-9** where median expressions of expressed cells of two isoforms are around 9.0 (log2 scale).

Figure S12: Distribution of isoform patterns in different sparsity levels of isoforms: **case 10-10** where median expressions of expressed cells of two isoforms are around 10.0 (log2 scale).

Figure S13: Distribution of isoform patterns in different sparsity levels of isoforms: case **7-6** where median expressions of expressed cells of two isoforms are fold-change of 2.0 different.

Figure S14: Distribution of isoform patterns in different sparsity levels of isoforms: **case 7-8** where median expressions of expressed cells of two isoforms are fold-change of 2.0 different.

Figure S15: Distribution of isoform patterns in different sparsity levels of isoforms: **case 7-5** where median expressions of expressed cells of two isoforms are fold-change of 4.0 different.

Figure S16: Distribution of isoform patterns in different sparsity levels of isoforms: **case 7-9** where median expressions of expressed cells of two isoforms have a fold-change of 4.0 different.

Figure S17: Distribution of isoform patterns in different sparsity levels of isoforms: **case 5-10** where median expressions of expressed cells of two isoforms have a significant fold-change of difference.

Figure S18: P-value distributions of the non-randomness tests from the simulated dataset *ipSim*.


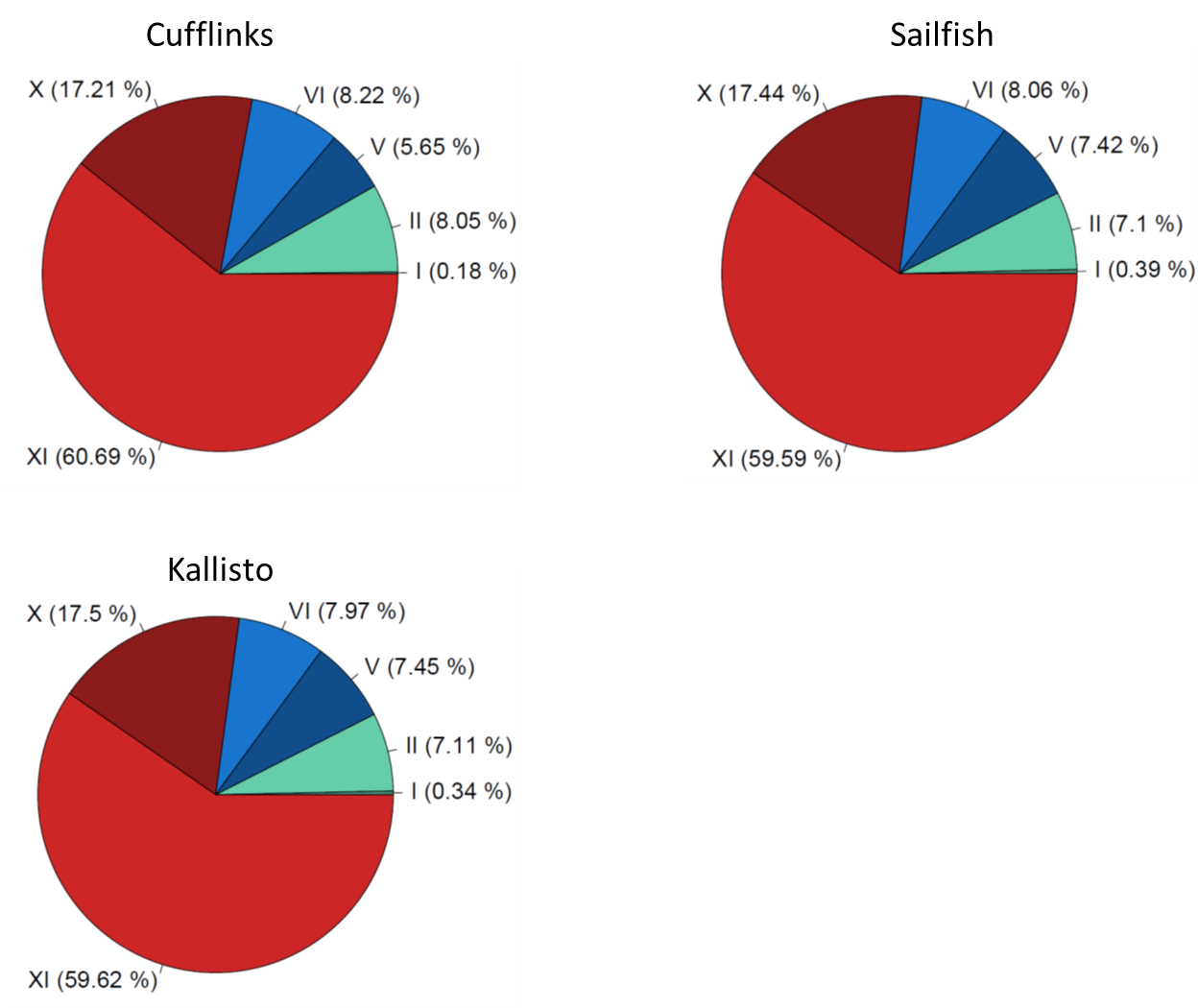


Figure S19: Distribution of isoform patterns from the MDA-MB-231 across three isoform quantification methods Cufflinks, Kallisto and Sailfish.

**References**

Law,C.W. *et al.* (2014) voom: Precision weights unlock linear model analysis tools for RNA-seq read counts. *Genome Biol.*, **15**, R29.

McCarthy,D.J. *et al.* (2012) Differential expression analysis of multifactor RNA-Seq experiments with respect to biological variation. *Nucleic Acids Res.*, **40**, 4288–4297.

Smyth,G.K. (2005) limma: Linear Models for Microarray Data. In, Gentleman,R. *et al.* (eds), *Bioinformatics and Computational Biology Solutions Using R and Bioconductor*, Statistics for Biology and Health. Springer New York, pp. 397–420.

Vu,T.N. *et al.* (2016) Beta-Poisson model for single-cell RNA-seq data analyses. *Bioinformatics*, btw202.
